# Pain Anticipation and Nocebo-Related Responses: A Descriptive Mini-Review of Functional Neuroimaging Studies in Normal Subjects and Precious Hints on Pain Processing in the Context of Neurodegenerative Disorders

**DOI:** 10.3389/fphar.2019.00969

**Published:** 2019-09-02

**Authors:** Martina Amanzio, Sara Palermo

**Affiliations:** ^1^Department of Psychology, University of Turin, Turin, Italy; ^2^European Innovation Partnership on Active and Healthy Ageing, Bruxelles, Belgium

**Keywords:** negative expectation, pain anticipation, fMRI, nocebo responses, prefrontal dorsolateral cortex, anterior cingulate cortex, anterior insular cortex

## Abstract

The exacerbation of a clinical condition or the occurrence of negative symptoms after an inert substance dispensation or a sham treatment is known as “nocebo effect.” Nocebo is the psychobiological effect due to the negative psychosocial context that accompanies a therapy, and it is a direct consequence of negative expectations by the patients and their own personal characteristics. Although the clinical relevance of the phenomenon is now recognized, a small number of studies have tried to ascertain its neural underpinnings (that it means nocebo responses). Moreover, there is no consensus on the brain networks involved in nocebo processes in humans. In particular, nocebo hyperalgesia has attracted almost no research attention. We conducted a mini-review on the few experimental pain functional magnetic resonance imaging (fMRI) studies of nocebo responses to discuss how negative expectancies and conditioning effects engage brain networks to modulate pain experiences. Moreover, we present possible clinical implications considering Alzheimer’s disease and behavioral frontotemporal dementia, in which the existence of a hypothetically disrupted neurocognitive anticipatory network—secondary to an endogenous pain modulatory system damage—may be responsible for pain processing alterations.

## Introduction

Understanding nocebo responses is important for both clinicians and neuroscientists, first of all, because it is substantial across disorders and could be associated with objective pathology and survival. Moreover, research on nocebo provides a way to investigate how the brain systems implicated in the processing of contextual information influence physiology and clinically relevant outcomes.

In the last two decades, functional neuroimaging studies analyzed the association among negative expectation, pain anticipation, and nocebo responses in humans. Brain imaging techniques have been invaluable to explore the neurobiology of pain anticipatory mechanisms occurring *via* inert substances and negative verbal suggestions when hyperalgesic stimuli are not dispensed. In such kind of circumstances, the experimenter presents the “supposed incoming pain” in such a believable way to induce the expectation of a “real painful stimulation.” Subjects’ psychobiological responses may be recorded in functional magnetic resonance imaging (fMRI) paradigms where the anticipatory phase of pain—considered as the time that elapses between the beginning of the neuroimaging acquisition and the beginning of the painful stimulation—is acquired.

Within this research framework, this mini-review first addresses the theme of neurofunctional pain anticipation mechanisms. The results of the only meta-analysis in the literature will be presented in order to summarize data from the literature in human pain experimental models using fMRI activation foci, and it will be used as an explanatory cue to interpret nocebo responses (Palermo et al., 2015). Indeed, our results revealed that pain anticipation may involve cortical systems implicated in the pain experience, even in the absence of a painful stimulation (Palermo et al., 2015). Moreover, the involved brain networks have a special role in selecting sensory (pain/interoception), attentional, and emotional resources (Palermo et al., 2015). Interestingly, this context emphasizes the need for a proper motivational attitude to predict potentially harmful events and consequently to trigger a cascade of cognitive control signals. The latter have the most important impact on how the potential harmful stimulus is processed in order to activate the motivation to escape from the incoming noxious event. An accurate processing of the negative psychosocial context and expectancies—responsible for the consequent activation of a cascade of neural responses at a cognitive-affective/motivational level—is fundamental for individual survival.

This mini-review also analyzes how negative expectancies and nocebo conditioning effect engage brain networks to modulate pain experiences in the few fMRI paradigms evaluating nocebo responses. In particular, the proposed fMRI studies not only analyzed the cortical–subcortical and spinal neuronal substrate associated with the conscious influence of negative expectancies in pain anticipation paradigms but also took into consideration the conditioned nocebo response. A model derived from a series of studies (Benedetti et al., 2003) describes nocebo in pain as a process necessarily mediated by conscious expectations. Such expectations can be induced not only by instructional/observational learning or verbal suggestion but also by classical conditioning (*Pavlovian conditioning*). The repeated association among the clinical context around the patient (for example, a syringe or the medical staff), the alleged pharmacological principle (the substance contained in the syringe), and an increased level of experienced pain usually induce a conditioned response, so after repeated associations, only the sight of the syringe or of the doctor will be sufficient to induce hyperalgesia.

Finally, we present possible clinical implications considering Alzheimer’s disease (AD) and frontotemporal dementia (FTD), where a failure of functional recruitment of cortical network corroborates an altered response to pain and a poor response to analgesic treatments (Defrin et al., 2016).

## Selection of Studies

A systematic search strategy was implemented to identify relevant studies on nocebo responses, published until 31 December 2018, across the online database most frequently used in the international literature (Medline database with PubMed literature search: http://www.ncbi.nlm.nih.gov/pubmed). We used a single set of query terms: *nocebo* AND *fMRI* [ALL]. With this aim, we reviewed the relevant literature in order to ensure the following: 1) the use of only inert substances or verbal suggestion (not studies on nocebo effects in the context of drug application); 2) the pain anticipation paradigm included a nocebo condition to study related functional activity; 3) the studies reported cerebral activation and deactivation changes, as assessed by blood-oxygen-level-dependent (BOLD) imaging; and 4) the experimental population was composed of healthy subjects. While we were in the selection phase, we found few studies that analyzed nocebo-related responses in the context of fMRI protocols, allowing us only to conduct a descriptive mini-review. The articles selected for the present mini-review are indicated in **Table 1**.

**Table 1:** fMRI studies that were included in the narrative review.

First author	Short details	URL	Pain stimuli	fMRI sample (nocebo group)
Icenhour A	*Neurogastroenterol Motil*. 2017	/pubmed/28177183	Visceral	16 healthy
Ellerbrock I	*Pain*. 2015	/pubmed/26181304	Heat	40 healthy
Schmid J	*Neuroimage*. 2015	/pubmed/26123378	Visceral	28 healthy
Freeman S	*Neuroimage*. 2015	/pubmed/25776211	Heat	24 healthy
Jensen KB	*Cereb Cortex*. 2015	/pubmed/25452576	Heat	24 healthy
Geuter S	*J Neurosci*. 2013	/pubmed/23966699	Capsaicin	20 healthy
Schmid J	*Pain*. 2013	/pubmed/23867733	Visceral	18 healthy
Kong J	*J Neurosci*. 2008	/pubmed/19052227	Heat	13 healthy

## Pain Anticipation

As we have previously reported (Palermo et al., 2015), “pain anticipation may have an important protective function, allowing the avoidance of bodily harm through the initiation of adaptive behavior essential for individual survival.” Only one study analyzed cerebral areas reliably involved in pain anticipation in humans through a coordinate‐based meta‐analysis approach (Palermo et al., 2015): *activation likelihood estimation* (ALE), which determines the convergence of foci reported from different experiments (Eickhoff et al., 2009; Salimi-Khorshidi et al., 2009). The authors provided an analysis of the fMRI literature assessing neuronal changes occurring during pain expectation in order to provide an empirical novel explanation of the cerebral networks that are consistently activated when a subject is anticipating a painful event to occur (Palermo et al., 2015). Moreover, the cited study (Palermo et al., 2015) further provided a meta‐analytic connectivity model (MACM) (Eickhoff et al., 2011; Laird et al., 2013), with the aim to explore the brain‐wide functional connectivity pattern of given ALE brain regions (Palermo et al., 2015).

In the course of pain anticipation, neural activations were discovered in the dorsolateral prefrontal, midcingulate, and anterior insula cortices; medial and inferior frontal gyri; inferior parietal lobule; middle and superior temporal gyri; and thalamus and caudate (Palermo et al., 2015). Deactivated foci were found in the anterior cingulate, superior frontal gyrus, parahippocampal gyrus, and claustrum (Palermo et al., 2015).

**Figure 1:**
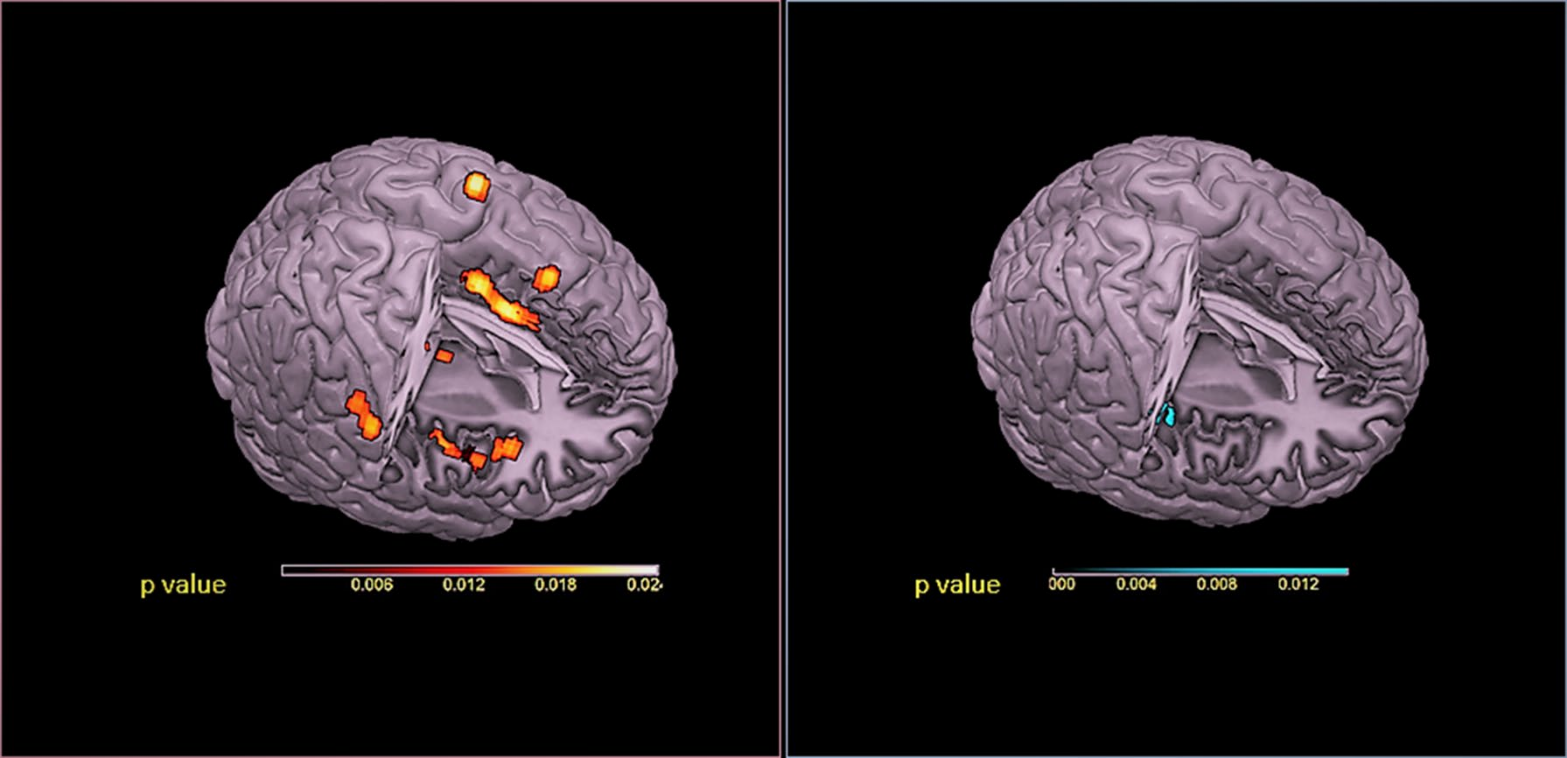
Red panel: Activations were projected onto a 3D rendering model of the brain. Blue panel: Deactivations were projected onto a 3D rendering model of the brain. ALE maps were computed using GingerALE 2.3.1 at a false discovery rate (FDR)-corrected threshold of P < 0.05, with a minimum cluster size of K > 50 mm^3^ and visualized using MRIcron.

Pain anticipation seems to involve cortical systems implicated in the experience of pain, even in the absence of a harmful stimulation, where a special role is played by the dorsal anterior cingulate cortex (dACC), anterior insular cortex (AIC), and lateral and medial prefrontal cortices (Palermo et al., 2015) (**Figure 1**).

The involvement of these key regions of the medial pain pathway (Petrovic et al., 1999; Rainville, 2002; Vogt, 2005)—previously implicated in unpleasant-affective dimensions of pain and the motivation to escape from a noxious event (Treede et al., 1999; Price, 2000)—suggested that these activations have a preparatory function for which potentially threatening stimuli receive more attention, thus supporting their reliable detection. Considering the above, in order to explore the behavioral domains associated with the functional connectivity network, MACM was applied on two seed regions (ACC and AIC) (Palermo et al., 2015). Interestingly, the selected seed regions produced very consistent results (Palermo et al., 2015). Moreover, activations were highly correlated to the behavioral domains of action (imagination, inhibition, and execution), emotion (fear), and perception (interoception and pain) (Palermo et al., 2015). We have suggested that pain anticipation paradigms activated a supramodal system where ACC and AIC enact a leading role in selecting sensory, emotional, and attentional resources (Palermo et al., 2015). Our MACM results seem to demonstrate an involvement of a dynamic process that facilitates the detection of fundamental environmental salient stimuli in the pain anticipation phenomena (Palermo et al., 2015). In particular, ACC and AIC may be considered—at a supramodal level—a hub connecting neural systems implicated in the depiction of the affective qualities of sensory events and interoceptive signals and in action monitoring (Palermo et al., 2015). Indeed, ACC and AIC play a distinctive role in embodying conceptual information relevant in order to transduce concepts into physiological and affective-behavioral responses and for survival (Roy et al., 2012).

In this direction, a study in women with genito-pelvic pain/penetration disorder demonstrated that pain anticipation modulatory neural activations—implicated in salience detection, emotion/arousal, autonomic responses, and executive functioning—may underlie increased levels of pain-related fear/anxiety and hypervigilance (Pazmany et al., 2017).

## Relationship Between Pain Anticipation, Negative Expectancies, and Nocebo Responses

Understanding how negative context and anticipatory expectancies influencing pain is essential for understanding nocebo responses. Indeed, nocebo hyperalgesia could be the result of negative outcome expectations leading to amplified pain experiences (Tracey, 2010). The findings have significance for chronic pain states and neurodegenerative disorders where abnormal functioning of specific brain areas might affect the analgesic outcome.

The first study that analyzed nocebo responses on healthy subjects used an inert treatment on the subjects believed to be hyperalgesic, thus creating an adversive pain expectancy experience (Kong et al., 2008). Kong and collaborators (Kong et al., 2008) demonstrated that subjective-rated pain intensity increased significantly more on nocebo regions compared with control regions in which no negative-expectation manipulation was performed. As reported by the authors (Kong et al., 2008), fMRI analysis of hyperalgesic nocebo responses to identical calibrated noxious stimuli showed signal increases in brain regions including “bilateral dorsal anterior cingulate cortex, insula, superior temporal gyrus, left frontal and parietal operculum, medial frontal gyrus, orbital prefrontal cortex, superior parietal lobule, and hippocampus; right claustrum/putamen, lateral prefrontal gyrus, and middle temporal gyrus.” Functional connectivity analysis of spontaneous resting-state indicated a correlation between the left frontal operculum and hippocampus (considered as seed regions) and pain network including the ACC, bilateral insula, operculum, and left primary somatosensory/motor cortices (Kong et al., 2008). Consequently, nocebo hyperalgesia may mostly turn out through a cognitive-affective pain pathway (the so-called medial pain system). Moreover, the left hippocampus may also be considerably involved in this process. The results were consistent with our previous study on pain anticipation (Palermo et al., 2015), with the exception for the hippocampus activation, probably due to the specificity of ALE meta-analysis, able to purge a limitation of individual fMRI studies (low power, methodological heterogeneity, and outcome discrepancies) (Eickhoff et al., 2009; Salimi-Khorshidi et al., 2009). Indeed, ALE meta-analysis accounts for these problems and draws more reliable inferences (Eickhoff et al., 2009; Salimi-Khorshidi et al., 2009) “by modelling the observed heterogeneity between studies, combining the available information to increase power and ultimately *separating the consistent findings from those that happened by chance*” (Samartsidis et al., 2017).

A further fMRI study on healthy subjects analyzed negative context information, inducing a nocebo manipulation through verbal suggestions (Ellerbrock et al., 2015). This study demonstrated how nocebo context not only modulated pain perception but also induced a specific operculum activation. Outstandingly, the operculum exhibited changes in coupling for the period of the nociceptive input, as proved by pinpoint differences and decreased connectivity with the basal ganglia, depending on whether a nocebo context has been provided or not (Ellerbrock et al., 2015). These findings suggested that negative verbal suggestions modulate not only cortical regions—as the previous study suggested (Kong et al., 2008)—but also basal ganglia–thalamocortical loops (Ellerbrock et al., 2015).

Another research, performed on healthy adults, highlighted how negative expectation can change pain experiences (Freeman et al., 2015). The authors investigated BOLD signal changes associated with administration of identical pain stimuli prior to and following the dispensation of the inert treatment (labelled as capsaicin) (Freeman et al., 2015). The findings highlighted that the expectation of a pain augmentation induced relevant neuronal modification and nocebo behavioral responses in the insula, orbitofrontal cortex, and periaqueductal gray (Freeman et al., 2015).

Nocebo responses were also analyzed through experimental visceral pain models in healthy volunteers. The first fMRI research analyzed nocebo responses as negative treatment expectations in rectal pain (Schmid et al., 2013). Painful rectal distensions have been produced following intravenous application of an inert substance combined with negative instructions of pain increase (nocebo experimental group). Neural activation during cued-pain anticipation and pain was analyzed along with expected and perceived pain intensity (Schmid et al., 2013). Negative expectations led to considerable insula hyperactivation during painful stimulation within the nocebo group. Moreover, direct group contrasts (nocebo versus placebo), during expectation modulation, revealed distension-induced somatosensory cortex hyperactivation in the nocebo group (Schmid et al., 2013). In a second research on this topic, greater increases in both expected and perceived pain were demonstrated (Schmid et al., 2015). Functional neuroimaging revealed that behavioral changes were associated with increased activation within the secondary somatosensory cortex and amygdala in nocebo responders during pain anticipation (Schmid et al., 2015). A subsequent psycho-physiological interaction analysis of the pain phase demonstrated increased functional connectivity between the selected seed region posed in the anterior insula and midcingulate cortex as a function of negative expectations (Schmid et al., 2015). The authors suggested that “negative pain-related expectations may play a crucial role in amplification of visceral pain, which could be mediated, at least in part, by a neural up-regulation of pain-associated areas and their own functional connectivity” (Schmid et al., 2015). In a third research, the authors explored neural and behavioral correlates of nocebo responses induced by classical conditioning (Icenhour et al., 2017). fMRI results showed alteration in the neural activation patterns during pain anticipation and visceral stimulation induced by prior conditioning (Icenhour et al., 2017). As reported by Icenhour and colleagues (Icenhour et al., 2017), “in the absence of behavioral effects, markedly altered neural responses may indicate conditioning involving altered attention, reappraisal, and perceptual acuity as processes contributing to the pathophysiology of visceral pain,” where somatosensory, medial prefrontal, and cingulate cortices and caudate play an important role. The study has to be considered crucial in investigating conditioned nocebo responses. Indeed, studies investigating the mechanisms underlying nocebo responses in pain focused primarily on negative cognitive expectations induced by verbal suggestions. However, nocebo effects can be interpreted as *a special case of contextual learning* in which previous context experiences are linked to corresponding responses, which can be automatic procedures with little flexibility or highly adaptive procedures modified by the associated contexts and consequences. Interestingly, placebo/nocebo effects can be an emblematic specimen of the combination of the two: (Palermo et al., 2015) *conditioning effect* (a fixed, instinctual, and automatic response) and (Benedetti et al., 2003) *cognitive expectancy effect* (a flexible adaptive response modified by predominant conscious context) (Kong and Benedetti, 2014). Considering the conditioning effect, only one fMRI study analyzed neural nocebo response mechanisms in nonconscious activation of conditioned pain responses (Jensen et al., 2015). The authors demonstrated that the human brain has a nonconscious mechanism to respond to conditioned cues (Jensen et al., 2015). Specifically, during nonconscious nocebo, activations of a network involving the thalamus, amygdala, and hippocampus were found (Jensen et al., 2015).

To conclude, the nocebo hyperalgesic effect may also envisage a pain-facilitating spinal cord mechanism at a very early stage of pain processing, well before cortical processing (Jensen et al., 2015). In this direction, an early enhancement of pain signals in the dorsal horn of the spinal cord has been found in a study in which nocebo hyperalgesia was investigated in combination with spinal fMRI in healthy volunteers (Geuter and Büchel, 2013). Indeed, pain stimulation induced a strong activation in the spinal cord at the level of the stimulated dermatomes C5/C6. Moreover, nocebo versus control condition contrast revealed enhanced nocebo-related activity in the ipsilateral dorsal horn (Geuter and Büchel, 2013).

## Pain Processing in the Context of Neurodegenerative Disorders

When considering pain processing in neurodegenerative disorders—especially in Alzheimer’s disease (AD) and behavioral frontotemporal dementia (bv-FTD)—a reduction in the motivational and affective components of pain is often described (Scherder et al., 2003). Indeed, the anatomo-pathological changes associated with these neurodegenerative disorders have significant effects on cognitive-affective and pain processing and also interfere with effective pain modulation (Palermo et al., 2015). Neurodegeneration also includes involvement of areas processing affective-motivational aspects of pain (i.e., the prefrontal cortex, ACC, insula, and amygdala). Since the prefrontal, the anterior cingulate, and the insula cortices appear to be elicited during pain anticipation (Palermo et al., 2015), it may be hypothesized a disruption of expectation-related mechanisms in this kind of patients.

If we consider the neuropathogenic changes related to the AD and bv-FTD onset, it is possible to notice that they involve the neural underpinnings of pain processing (Carlino et al., 2010; Monroe et al., 2012): In particular, the perception of pain and the neural circuits associated with its behavioral expression may be hyperactive/hypoactive, depending on the neural region involved and the severity of the neurodegeneration. Considering the above, we hypothesize that also pain anticipation processing and nocebo responses could be short-circuited in neurodegenerative disorders. Indeed, functional neuroimaging can potentially fill the gaps in our knowledge of these phenomena in AD and bv-FTD. In particular, since AD and bv-FTD patients are likely to show medial prefrontal cortex impairment—also related to the target constituents of the medial pain system and to the neuroanatomical localization of cognitive pain-expectation-related mechanisms, we hypothesize that an impaired “action monitoring” may be considered an important factor to be addressed (Amanzio et al., 2016; Amanzio et al., 2017a; Amanzio et al., 2017b; Amanzio et al., 2018).

Although no studies exist concerning pain anticipation in AD and bv-FTD due to ethical constraints, it is significant to report a study on pain anticipation in mild traumatic brain injury (TBI) (Strigo et al., 2014). When compared with normal controls, TBI subjects showed increased activation within the midbrain periaqueductal grey, the right dorsolateral prefrontal cortex, and the left cuneus (Strigo et al., 2014). These findings suggested that mild TBI can negatively affect anticipatory pain processing and interferes with effective pain modulation (Strigo et al., 2014). Indeed, the authors proposed a speculative model for which a potentially disrupted neurocognitive anticipatory network—secondary to a endogenous pain modulatory system damage—exists and underlies difficulties in pain processing regulation (ဂStrigo et al., 2014).

## Clinical Implications

**Figure 2:**
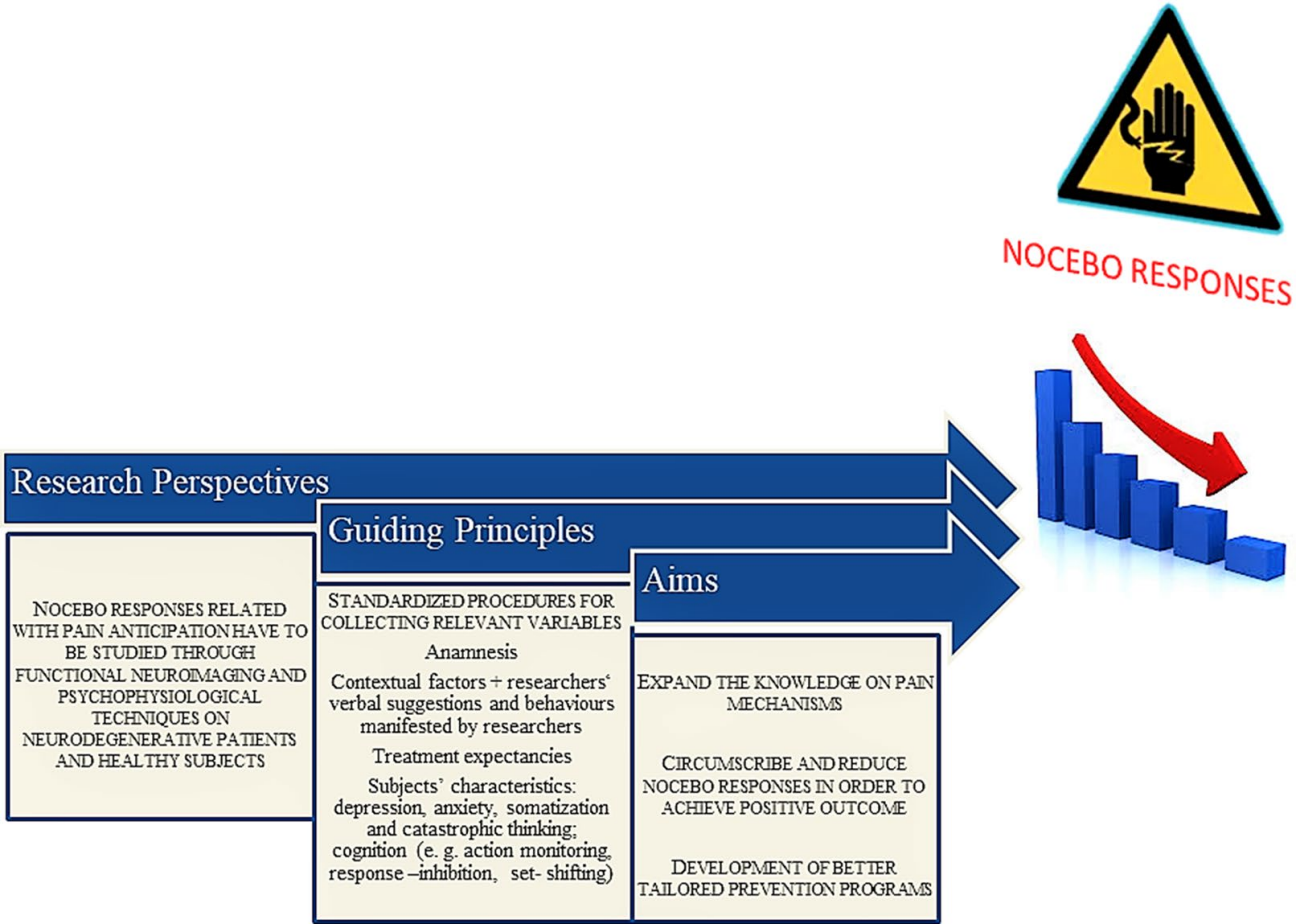
A suggested stepwise theoretical algorithm to study pain anticipation and nocebo-related responses.

The neurobiological basis of the nocebo effect is only now beginning to be disentangled. One of the most productive models to better understand the neurobiology of the nocebo effect is pain. Pain anticipation and nocebo response neural correlates have been studied with fMRI neuroimaging techniques in the last decade. A host of cortical and subcortical regions can be activated by various negative verbal instructions and contexts. Those areas identified a set of core sites related with an affective-cognitive pain pathway where the prefrontal, cingulate, insular, and orbital cortices play an important role. Other areas less reported in the literature include the brainstem, hippocampus, amygdala, superior temporal gyrus, parietal operculum, and superior parietal lobule. Nocebo effects are clinically significant but are often underrecognized in clinical practice (Chavarria et al., 2017). Moreover, they are very prevalent among neurological diseases, resulting in low adherence and treatment outcome (Chamsi-Pasha et al., 2017). The objective of future studies will be to manage, circumscribe, and possibly reduce nocebo responses as a vulnerable point that should be minimized (see **Figure 2**).

Indeed, the current clinical approach is to maximize the placebo effect and minimize the nocebo effect to provide the greatest possible benefit to the patient (Chavarria et al., 2017; Evers et al., 2018). These studies will present important challenges a) to recognize cerebral biomarkers and predispose psychological and neuropsychological factors; b) to recognize the nocebo-driven adverse effects; c) to identify individuals more prone to develop nocebo effects; and d) to identify effective strategies to minimize nocebo (Data-Franco and Berk, 2013).

Studies in the field of pain have previously proposed three different mechanisms to explain the nocebo effect: expectation, conditioning, and anticipatory anxiety (Chamsi-Pasha et al., 2017). Taking into account these three mechanisms of action, it is possible to improve the clinical outcome by putting the following factors into play (Evers et al., 2018; Hansen and Zech, 2019): knowledge and recognition of nocebo effects; addition of meaning to the transmitted information and use of positive suggestions and clinical examples; neutralization of negative expectations and avoidance of generation of new negative expectations; utilization of the focused attention and suggestibility of the stress-induced natural trance state; and development of a trusting and encouraging therapeutic relationship. Moreover, administering patients’ past experiences and beliefs are at the basis of possible strategies (Chavarria et al., 2017).

For example, doctors usually warn patients of the painful nature of an impending procedure (which means “pain anticipation” in the patients’ mind). This may have an iatrogenic effect and may increase the pain subjectively experienced by the patient at the time of treatment administration. Indeed, it has been demonstrated that lower pain scores occur if the message was focused on the treatment’s beneficial effects rather than it being painful (Bishop et al., 2017).

There is no doubt that “only with attention to the empirical findings from programmatic research of specific and nonspecific effects and their interaction is it possible to improve the outcomes of treatment beyond the status quo” (Bootzin and Bailey, 2005).

## Conclusions

In our mini-review, we consider the systems-level neurobiology that underlies nocebo effects; we focus primarily on pain, which has been most extensively studied. The issues of nocebo responses basically encompass both the site and the mode of brain function. It can be considered as the initial stage in the genesis of pain perception and conscious suffering. A better understanding of the nocebo-related responses is crucial to relieve its impact on clinical practice and to enhance the therapeutic outcome. The above statements have relevance for neurodegenerative disorders and chronic pain states, for which the functioning of essential brain regions related with nocebo responses may amplify negative outcome to treatments through an activation of a neural cognitive-affective network.

## Author Contributions

MA conceived the content of the review, wrote the first draft of the manuscript, supervised subsequent changes, and took part in critique processes. SP wrote the manuscript, produced images and tables, and took part in the review and critique processes.

## Conflict of Interest Statement

The authors declare that the research was conducted in the absence of any commercial or financial relationships that could be construed as a potential conflict of interest.

## Funding

This work was supported by the fund GEMG_RILO_18.
